# Lung Cancer in Pregnancy: An Unusual Case of Complete Response to Chemotherapy

**DOI:** 10.7759/cureus.440

**Published:** 2015-12-29

**Authors:** Ryan Yates, Jun Zhang

**Affiliations:** 1 Medicine, Baylor College of Medicine

**Keywords:** lung cancer, pregnancy, lymphoepithelioma-like, squamous cell carcinoma, platinum-based chemotherapy

## Abstract

The diagnosis of lung cancer in pregnancy is rare. Most cases are quite advanced and have dismal outcomes despite treatment. We present the case of a 26-year-old woman who was diagnosed with Stage IIIA (T3N2M0) squamous-cell carcinoma of the lung with lymphoepithelioma-like features at the 18^th^ week of pregnancy. A chest CT revealed a large right hilar mass with obliteration of the right main bronchus and resulting collapse of the right lung with mediastinal shift to the right. A transbronchial biopsy of the mass and a subcarinal lymph node confirmed poorly differentiated squamous cell carcinoma with lymphoepithelioma-like features. Brain MRI, PET, and CT scans were negative for distant metastasis. The patient received four cycles of neoadjuvant cisplatin and docetaxel with a complete radiographic response. She delivered a healthy baby girl at 35 weeks gestation. Post-partum, she received radiation to the right hilum and mediastinum as consolidation. The patient continues to remain free of disease more than 16 months after initial diagnosis. To our knowledge, this is the only reported case of lung cancer in pregnancy where there is a complete response to chemotherapy. The histology is also distinct from other reported cases. In addition, this case exemplifies the relative safety and efficacy of chemotherapy during the later stages of pregnancy. As long as a patient is beyond the first trimester of pregnancy, platinum-based doublet chemotherapy may be considered as a feasible treatment option.

## Introduction

Cancer in pregnancy is rare, affecting approximately 1/1000 term pregnancies. Breast cancer, cervical cancer, leukemia, lymphoma, and melanoma are the most common malignancies diagnosed in pregnancy [1-2]. Lung cancer is much rarer, with only 60 cases having been reported in the English language at the time of a 2013 case series [3]. Of the cases reported, the majority of women present with advanced lung cancer (Stage III or IV) and have very poor outcomes. The most frequent histology reported is adenocarcinoma [4].

Primary lymphoepithelioma-like carcinoma (LELC) of the lung is a distinct histologic entity that resembles nasopharyngeal carcinoma. First reported in 1987, it is classified as a type of non-small cell lung cancer (NSCLC), more specifically a variant of large cell carcinoma [5,7]. Most cases have been reported in Asian non-smokers and are closely associated with Epstein-Barr Virus (EBV) infection, although this association is variable. Patients with advanced disease are often treated with a combination of chemotherapy and radiation [6]. To our knowledge, there have been no reported cases of primary LELC of the lung in a pregnant patient.

The treatment of lung cancer during gestation is quite challenging, as the risks and benefits to the mother and fetus are weighed. Limited experience and case reports suggest chemotherapy in the second and third trimester is both safe and efficacious [1-3].

We present the case of a 26-year-old African-American female diagnosed at 18 weeks gestation with Stage IIIA poorly differentiated squamous cell carcinoma of the lung with lymphoepithelioma-like features. She achieved a complete response to neoadjuvant chemotherapy during the second and third trimesters of pregnancy. She delivered a healthy baby girl at 35 weeks gestation and then underwent consolidative radiation. The patient remains disease-free 16 months after initial diagnosis, and the baby girl is healthy without any known abnormalities.

## Case presentation

**Figure 1:**
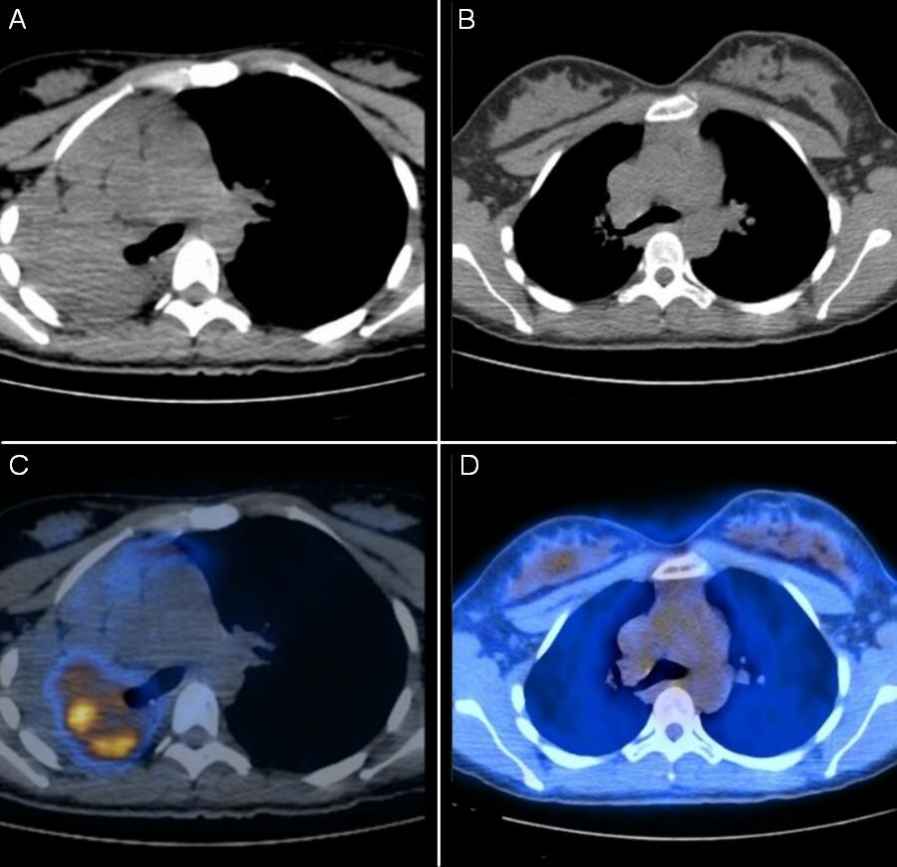
Pre-treatment and post-treatment images Pre-treatment CT chest (A) and fused PET/CT image (C) show large right hilar mass that obstructs the right main bronchus resulting in collapse of the right lung and mediastinal shift to the right. Post-treatment CT Chest (B) and fused PET/CT image (D) show complete radiographic response after treatment with chemotherapy.

A 26-year-old African-American female non-smoker with no significant past medical history presented at 18 weeks gestation with hemoptysis and dyspnea on exertion. Radiographs showed complete opacification of the right hemithorax with mediastinal shift to the right. CT scan showed a 6.7 x 4.7 cm right hilar mass with obliteration of the right main bronchus leading to a collapse of the right lung (Figure 1A).

**Figure 2:**
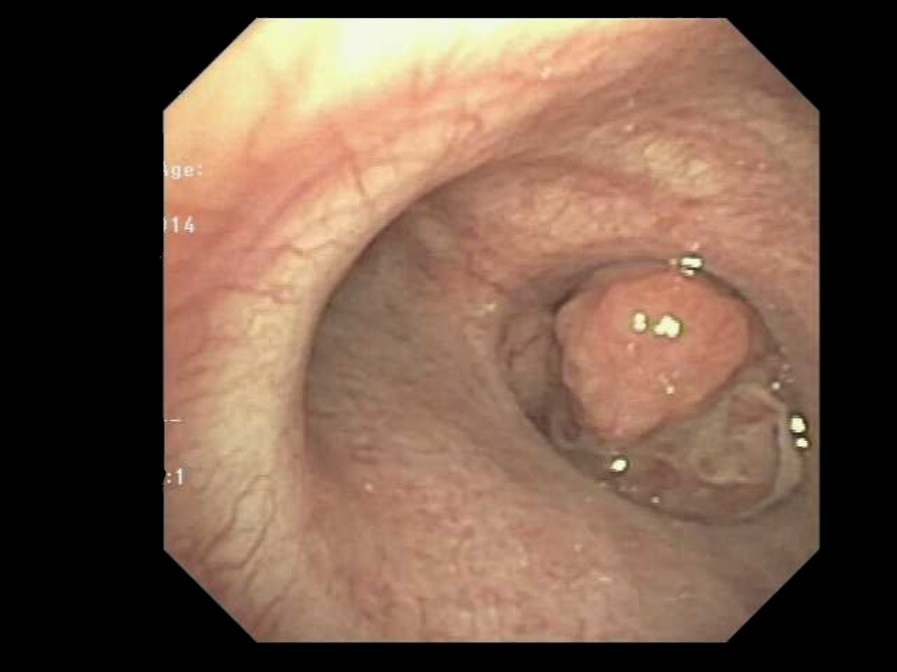
Bronchoscopic view of tumor protruding out of the right main bronchus

**Figure 3:**
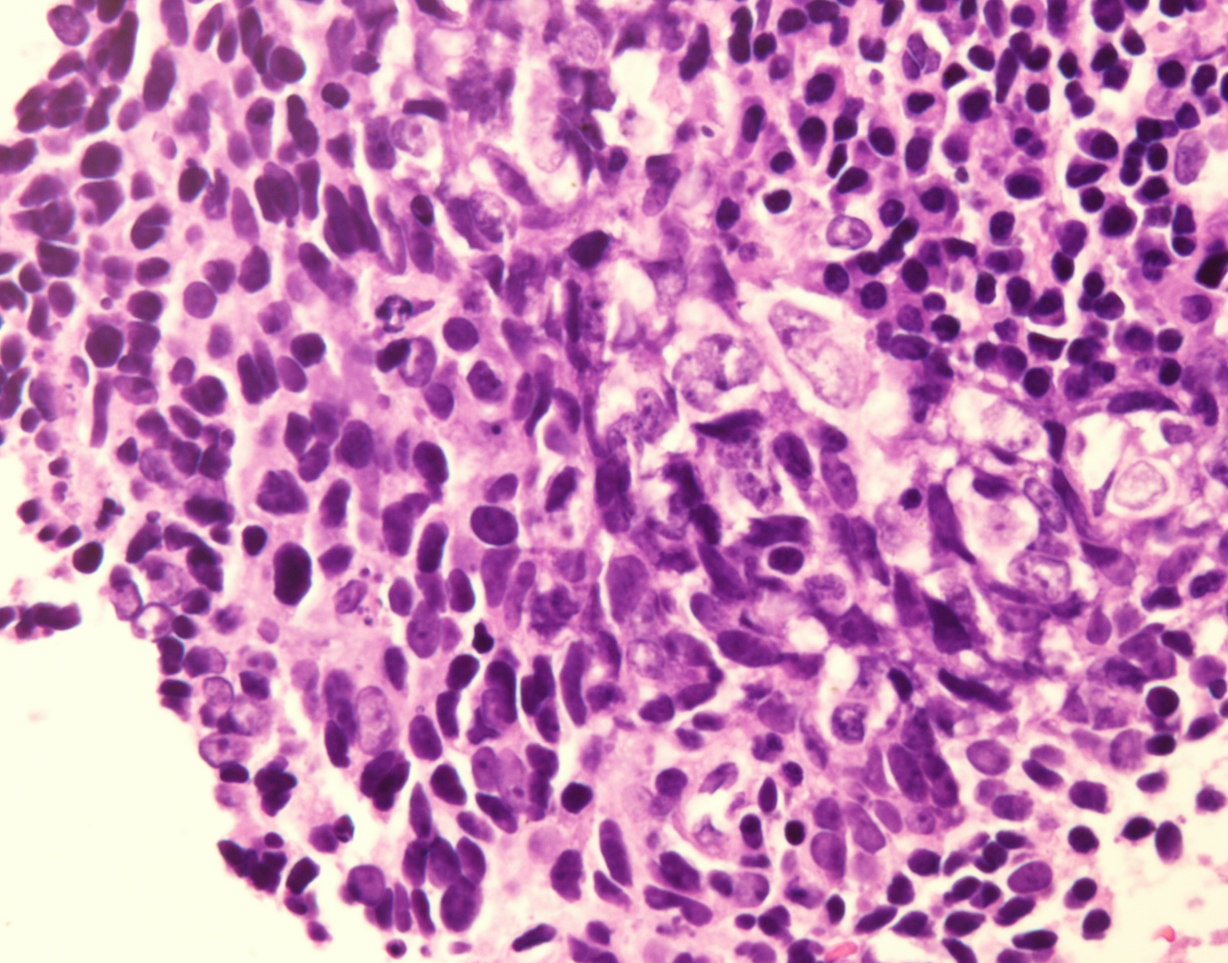
Hematoxylin- and eosin-stained specimen (400x) showing large, malignant tumor cells with scant cytoplasm scattered among a lymphoplasmacytic infiltrate

She underwent a laryngoscopy and a bronchoscopy, which revealed an endobronchial lesion in the right main bronchus (Figure 2). The lesion was friable when manipulated and extended up to but did not invade the carina. The bronchoscope was unable to be passed beyond the lesion. Multiple biopsies were obtained, and pathology confirmed poorly differentiated squamous cell carcinoma with lymphoepithelioma-like features (Figure 3). Immunohistochemistry was positive for keratin, vimentin, p63, and AE1/AE3 and negative for TTF-1, synaptophysin, chromogranin, and CK 5/6. Immunohistochemistry and in situ hybridization were negative for EBV.

PET and CT scans showed a large mass (5.5 x 6.0 x 7 cm) that surrounded and occluded the right main bronchus and extended into the subcarinal region with maximum standardized uptake value of 18 (Figure 1C). There was no evidence of distant metastatic disease on the PET, CT or brain MRI. After multidisciplinary discussion, an endobronchial ultrasound (EBUS) was done for mediastinal staging due to the consideration of definitive surgery after neoadjuvant chemotherapy. Biopsy of a subcarinal lymph node was positive for metastasis. The primary tumor was adherent to the wall of the right main stem bronchus with no tissue plane seen between the endoluminal portion of the tumor and the bronchial wall. The mass did not directly invade the carina. Stage IIIA, T3N2M0, squamous cell lung cancer was confirmed.

The patient was 23 weeks pregnant when she began chemotherapy. She received chemotherapy with cisplatin (75 mg/m^2^) and docetaxel (75 mg/m^2^) once every three weeks for four cycles. After the first cycle of chemotherapy, she was briefly hospitalized due to recurrence of hemoptysis, but it was milder than initial presentation and resolved spontaneously. She had no problems tolerating the subsequent cycles of chemotherapy. After two cycles of chemotherapy, restaging scans  showed an excellent response with restored patency of right main bronchus and aeration of the right lung. Mass had decreased in size to 4.1 x 3.0 x 5.4 cm.

The fetus was followed closely by maternal-fetal medicine specialists throughout the course of chemotherapy. There were no obvious signs of fetal distress. Three weeks after the patient’s fourth cycle of chemotherapy (at 35 weeks gestation), labor was induced, and the patient vaginally delivered a healthy, 2166 gram female. Apgar scores were 8 and 9 at 1 and 5 minutes, respectively.

A chest CT taken after delivery showed a further decrease in size of the right hilar mass, and PET and CT scans confirmed no metabolic activity in the mass, confirming a complete radiographic response to treatment (Figures 1B, 1D). A bronchoscopy with EBUS was performed once again, and multiple biopsies were negative for malignancy. Thoracic surgery re-evaluated the patient for potential right pneumonectomy, but the patient opted not to undergo surgery and instead decided to proceed with consolidative radiation. She received 60 Gy in 30 fractions to the right hilum and mediastinum. She subsequently developed grade 2 radiation pneumonitis requiring steroids. This improved, and steroids were eventually weaned off. To date (16 months after diagnosis), the patient remains without evidence of disease.

## Discussion

A 2013 case series analyzed 60 cases of lung cancer in pregnancy reported in the literature since 1953. Of these, the median age at diagnosis was 38.3 years, and median gestational age at diagnosis was 25.5 weeks. Only one of the cases was diagnosed as early stage (Stage I or II). Histology was NSCLC (mostly adenocarcinoma) in 80% of the cases, and only 22% of the patients received chemotherapy during gestation. Fetal outcomes were generally good with more than 80% reported as normal, but maternal outcomes were much more dismal, with only 19% of the patients alive at 12 months or more [3]. This case illustrates the relative safety and efficacy of combination cisplatin and docetaxel in gestational lung cancer. It is the only reported case that we are aware of in which a patient with advanced lung cancer achieves a complete response to chemotherapy administered during pregnancy.

To our knowledge, this is also the only reported case of lung cancer in pregnancy with lymphoepithelioma-like features. While primary LELC of the lung is a distinct histologic entity recognized by the World Health Organization, the pathologic specimens in our case shared many of the same histologic features. Tumors typically consist of solid nests of undifferentiated tumor cells in a syncytial arrangement surrounded by heavy lymphoplasmacytic infiltrate (Figure 3). Staining for cytokeratins and P63 is typically positive [6-7]. Also, while EBV is commonly associated with primary LELC of the lung in Asian patients, it does not appear to be as strongly associated in Western patients, as tumor cells in all 6 patients included in a 2001 review stained negative for EBV [8].

Primary LELC of the lung appears to be quite sensitive to chemotherapy and radiation, and the prognosis is thought to be better than other subtypes of NSCLC [7]. Perhaps the excellent response to chemotherapy seen in this patient is in part related to the lymphoepithelioma-like features present in her tumor.

Most of the evidence regarding safety and efficacy of chemotherapy during gestation comes from the breast cancer literature; however, a systematic review on the subject was published in 2010 that included eight women with lung cancer. All were treated with platinum-based regimens except one patient who received erlotinib. Fetal adverse events in this review were restricted to transient respiratory distress, which was attributed more to prematurity than to the chemotherapy administered during gestation. Regimens used included cisplatin or carboplatin in combination with paclitaxel, docetaxel, vinorelbine, gemcitabine, or etoposide [9].

Adverse fetal effects and congenital malformations are known to be increased if chemotherapy is administered during the first trimester of pregnancy; however, when chemotherapy is administered after the first trimester, the risk of congenital malformation appears to be no different than in the normal population. The most common adverse effects are growth restriction and premature delivery [2]. Other than low birth weight, there were no obvious adverse fetal effects in our case.

Finally, definitive radiation is contraindicated during pregnancy; however, palliative radiation to distant disease sites may be considered [3,10]. As there were no distant sites of disease in our patient, radiation was deferred until after delivery of the baby. The radiation likely contributed to the durable response seen.

## Conclusions

Although abundant evidence is limited in advanced gestational lung cancer, platinum-based cytotoxic chemotherapy may be a reasonable treatment option in patients who are beyond the first trimester. Specifically, this case illustrates the relative safety and efficacy of cisplatin and docetaxel combination chemotherapy for this group of patients.
